# Nontypeable *Haemophilus influenzae* Has Evolved Preferential Use of *N-*Acetylneuraminic Acid as a Host Adaptation

**DOI:** 10.1128/mBio.00422-19

**Published:** 2019-05-07

**Authors:** Preston S. K. Ng, Christopher J. Day, John M. Atack, Lauren E. Hartley-Tassell, Linda E. Winter, Tal Marshanski, Vered Padler-Karavani, Ajit Varki, Stephen J. Barenkamp, Michael A. Apicella, Michael P. Jennings

**Affiliations:** a Institute for Glycomics, Griffith University, Gold Coast, Queensland, Australia; b Department of Pediatrics, Saint Louis University School of Medicine, Saint Louis, Missouri, USA; c Cardinal Glennon Children’s Medical Center, Pediatric Research Institute, Saint Louis, Missouri, USA; d Department of Cell Research and Immunology, The George S. Wise Faculty of Life Sciences, Tel-Aviv University, Tel Aviv, Israel; e Glycobiology Research and Training Center, University of California, San Diego, San Diego, California, USA; f Department of Microbiology, Carver College of Medicine, University of Iowa, Iowa City, Iowa, USA; University of Mississippi Medical Center; University of Mississippi Medical Center

**Keywords:** Haemophilus influenzae, bacterial metabolism, glycobiology, microbial pathogenesis, sialic acid

## Abstract

Nontypeable *Haemophilus inﬂuenzae* (NTHi) is a Gram-negative bacterial pathogen that is adapted exclusively to human hosts. NTHi utilizes sialic acid from the host as a carbon source and as a terminal sugar on the outer membrane glycolipid lipooligosaccharide (LOS). Sialic acid expressed on LOS is critical in NTHi biofilm formation and immune evasion. There are two major forms of sialic acids in most mammals, *N*-acetylneuraminic acid (Neu5Ac) and *N*-glycolylneuraminic acid (Neu5Gc), the latter of which is derived from Neu5Ac. Humans lack the enzyme to convert Neu5Ac to Neu5Gc and do not express Neu5Gc in normal tissues; instead, Neu5Gc is recognized as a foreign antigen. A recent study showed that dietary Neu5Gc can be acquired by NTHi colonizing humans and then presented on LOS, which acts as an antigen for the initial induction of anti-Neu5Gc antibodies. Here we examined Neu5Gc uptake and presentation on NTHi LOS. We show that, although Neu5Gc and Neu5Ac are utilized equally well as sole carbon sources, Neu5Gc is not incorporated efficiently into LOS. When equal amounts of Neu5Gc and Neu5Ac are provided in culture media, there is ∼4-fold more Neu5Ac incorporated into LOS, suggesting a bias in a step of the LOS biosynthetic pathway. CMP-Neu5Ac synthetase (SiaB) was shown to have ∼4,000-fold-higher catalytic efficiency for Neu5Ac than for Neu5Gc. These data suggest that NTHi has adapted preferential utilization of Neu5Ac, thus avoiding presentation of the nonhuman Neu5Gc in the bacterial cell surface. The selective pressure for this adaptation may represent the human antibody response to the Neu5Gc xenoantigen.

## Abstract (precis)

Host-adapted bacterial pathogens such as NTHi cannot survive out of their host environment and have evolved host-specific mechanisms to obtain nutrients and evade the immune response. Relatively few of these host adaptations have been characterized at the molecular level. NTHi utilizes sialic acid as a nutrient and also incorporates this sugar into LOS, which is important in biofilm formation and immune evasion. In the present study, we showed that NTHi has evolved to preferentially utilize the Neu5Ac form of sialic acid. This adaptation is due to the substrate preference of the enzyme CMP-Neu5Ac synthetase, which synthesizes the activated form of Neu5Ac for macromolecule biosynthesis. This adaptation allows NTHi to evade killing by a human antibody response against the nonhuman sialic acid Neu5Gc.

## INTRODUCTION

Haemophilus influenzae is a host-adapted human pathogen that is categorized into typeable strains that express a polysaccharide capsule (serotypes a to f) and nontypeable (noncapsulated) H. influenzae (NTHi) strains (1). H. influenzae is carried asymptomatically in the upper respiratory tract of 40% to 80% of healthy humans (2). Capsulated H. influenzae strains typically cause invasive diseases such as meningitis, while NTHi strains are responsible for acute and chronic infections of the respiratory tract, such as middle ear infection in children (3), exacerbations of chronic obstructive pulmonary disease (COPD) in the elderly (4), and community-acquired pneumonia (5). Since the introduction of a vaccine against H. influenzae serotype b (Hib), the incidence of invasive infection caused by NTHi has increased significantly worldwide (6, 7). NTHi is now a major cause of severe invasive disease in neonates and in children who have significant comorbidities (8, 9). Invasive NTHi infections are fatal in ∼10% of children between 2 and 4 years of age and in ∼17% of children under the age of 1 (10, 11). The increase in invasive disease caused by NTHi is likely due to many factors, including increased numbers of vulnerable patient populations, rather than being solely due to Hib vaccine-induced strain replacement (6).

**FIG 1:**
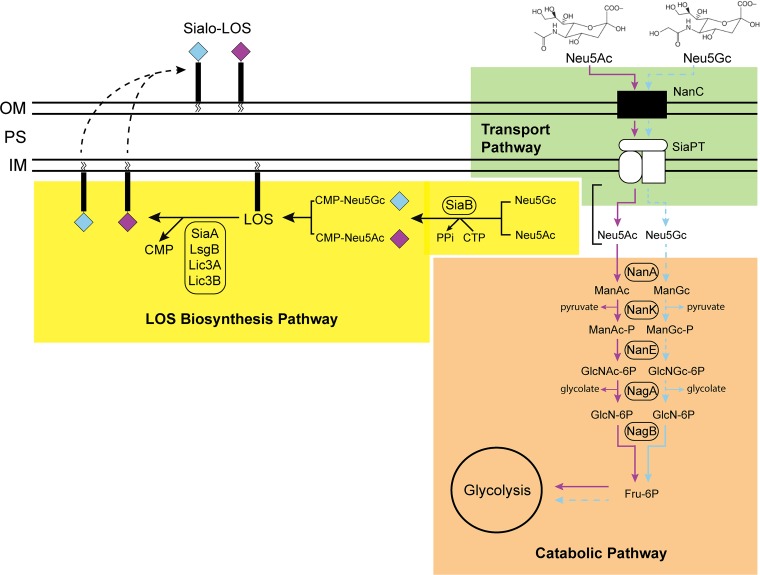
Overview of the sialic acid catabolic and LOS biosynthesis pathways of NTHi for sialic acids Neu5Ac (purple) and Neu5Gc (blue). Depending on its needs, NTHi can negate toxic building up of sialic acid by funneling excess sugar through the catabolic pathway or can convert sialic acid to an activated state for LOS sialylation. On the inner membrane (IM), the biosynthesis pathway converts sialic acid to CMP-sialic acid, which acts as an electron donor to drive the transfer of sialic acids as terminal sugars to LOS. The sialylated LOS is flipped through the periplasmic space (PS) onto the outer membrane (OM).

Sialic acids are a diverse group of carboxylated nine carbon-backbone sugars (12, 13) with *N*-glycolylneuraminic acid (Neu5Gc) and its precursor *N*-acetylneuraminic acid (Neu5Ac) being the two most abundant sialic acids found on the mammalian cell surface. Neu5Gc is present in many mammals, including the great apes, with one notable exception being humans (14, 15), owing to a mutation in the CMP-Neu5Ac hydroxylase enzyme CMAH (16–19), with the result that humans produce only Neu5Ac. Absence of Neu5Gc in humans was first discovered after investigation of the basis for “serum sickness,” a condition that results from the presence of Hanganutziu-Deicher (HD) antibodies (anti-Neu5Gc antibodies), which recognize Neu5Gc antigens in animal serum administered to human patients (20–22). Neu5Gc is present at very low or nondetectable levels in normal human body fluids and on tissue surfaces (18) but can be found in certain cancers (23–29), and anti-Neu5Gc antibodies have been proposed to be a biomarker to detect cancer (30, 31). However, recent studies have revealed Neu5Gc to be present in or on more human tissue types than initially thought, such as vascular endothelium (32), carcinomas (33), placental tissues (34), epithelial cells lining body organs (27), gangliosides (35) and on endothelia during vascular inflammation leading to atherosclerosis (32). It is thought that Neu5Gc from dietary sources such as red meat and dairy products is the source of this xenosugar on normal human tissue (27, 36). However, incorporation of Neu5Gc onto the cell surface has been demonstrated to mediate chronic inflammation as a result of the presence of anti-Neu5Gc antibodies in human serum, causing “xenosialitis” (37, 38). The source of these anti-Neu5Gc antibodies has been proposed to be NTHi colonization during infancy (39), as NTHi is able to decorate the termini of its LOS with sialic acids. This molecular mimicry of the host allows immune evasion (40, 41) and is also required for virulence (42) and biofilm formation (41, 43). NTHi cannot synthesize sialic acids and must scavenge exogenous sialic acid from the host (44). If Neu5Gc is present in the host from the diet, it may be acquired by NTHi along with Neu5Ac and either be incorporated into the LOS through the LOS biosynthetic pathway via the CMP-Neu5Ac synthetase, namely, sialic acid synthetase (SiaB), or serve as a carbon source via the catabolic pathway encoded by the *nan* genes (Fig. 1) (45).

In the present study, we examined the uptake and presentation of Neu5Gc and Neu5Ac on the NTHi cell surface to better understand the mechanism of NTHi induction of anti-Neu5Gc antibodies in humans.

## RESULTS

### Neu5Gc is efficiently utilized as a carbon source.

**FIG 2:**
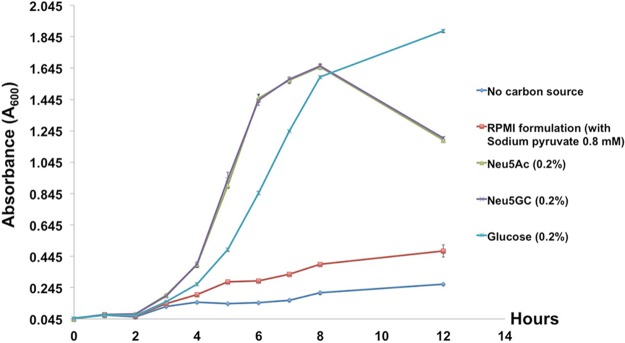
Growth of NTHi strain 2019 in sialic acid-free RPMI media. The medium was supplemented with a sole carbon source, and bacterial growth was monitored for 12 h. The growth curves of Neu5Ac and Neu5Gc were found to be nearly identical and are superimposed.

The utilization of Neu5Ac and its metabolic fate have been characterized in NTHi previously (46), but a study of the utilization of Neu5Gc by NTHi has not yet been carried out, although NTHi has been proposed to be a key antigen in the generation of anti-Neu5Gc antibodies (39). It was proposed that generation of these anti-Neu5Gc antibodies against NTHi requires incorporation of Neu5Gc from human serum acquired from the diet into the LOS by NTHi (39), likely through promiscuity of the LOS biosynthesis machinery or catabolic pathway (Fig. 1). We hypothesized that there may be a bias in sialic acid utilization by NTHi that drives incorporation of the relatively scarce Neu5Gc into NTHi LOS. For example, in cases of inefficient catabolism of Neu5Gc as a carbon source (relative to Neu5Ac), the lower rate of flux may generate a pool of Neu5Gc that is available for activation by addition of CMP and may thereby drive its preferential incorporation into LOS (Fig. 1). In fact, growth of NTHi strain 2019 using sialic acid-free chemically defined RPMI 1640 medium supplemented with 1% (vol/vol) hemin and 20 µg/ml NAD (sRPMI medium) (47) and supplemented with either Neu5Ac or Neu5Gc as the sole carbon source revealed that there was no difference in the growth rates of NTHi on these distinct sialic acids (Fig. 2).

### Preferential addition of Neu5Ac or Neu5Gc on NTHi LOS.

**FIG 3:**
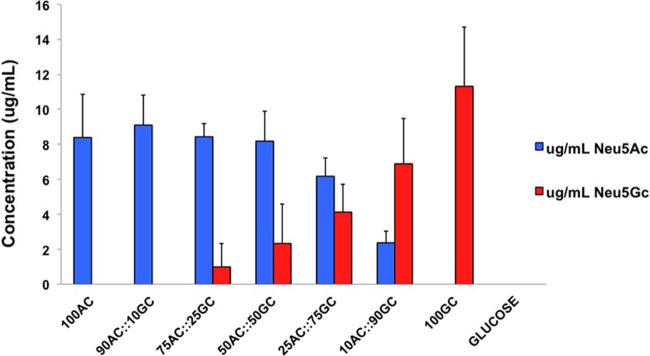
Quantified sialic acid (indicated in micrograms per milliliter) of highly pure NTHi LOS grown on RPMI media supplemented with various molarity ratios of Neu5Ac and Neu5Gc. At a 1:1 ratio, the amount of Neu5Ac measured was found to be significantly (4-fold) greater than that of Neu5Gc (*n* = 3, *P* = 0.0210). The *P* value was calculated using a two-tailed unpaired Student's *t* test.

On the basis of the results presented in Fig. 2, we concluded that Neu5Ac and Neu5Gc are utilized equally well by NTHi as a sole carbon source, and previous work demonstrated that the sialic acid transporter SiaP bound Neu5Ac and Neu5Gc with similar affinities (48). This indicated that the transport and catabolic pathways were unbiased in the utilization of these two sialic acids (Fig. 1). To investigate the potential for bias in the incorporation of Neu5Ac or Neu5Gc in the LOS biosynthesis pathway, NTHi strain 2019 was grown on sialic acid-free sRPMI media supplemented with various molar ratios of Neu5Ac and Neu5Gc (Fig. 3). NTHi LOS was purified from these cultures, and the amount of Neu5Ac and Neu5Gc present on LOS was determined by sugar analysis using high performance anion-exchange chromatography with amperometric detection. These results demonstrated a clear preference for incorporation of Neu5Ac over Neu5Gc. For example, when an equal 50:50 ratio of Neu5Ac/Neu5Gc was provided, 4-fold more Neu5Ac was present in LOS than Neu5Gc (*n* = 3; *P = *0.0210 [using Student's *t* test]).

### Preference for sialic acid Neu5Ac of NTHi is due to CMP-Neu5Ac synthetase (SiaB).

**FIG 4:**
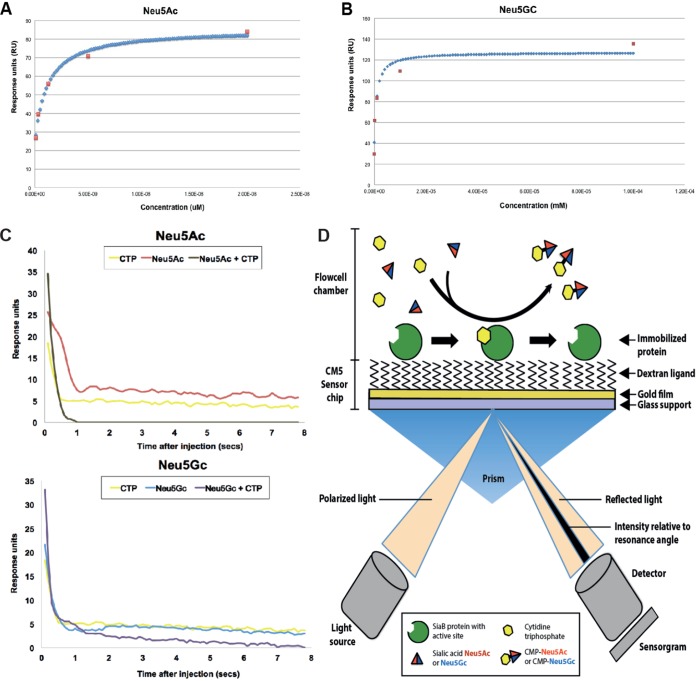
Enzyme kinetic measurements of NTHi SiaB (CMP-Neu5Ac synthase) enzyme determined using glycan array and surface plasmon resonance. (A) The *K_D_* value against sialic acid Neu5Ac was calculated to be 1.2 nM. Red dots represent dilution concentrations. Blue dots represent the best-fit curve. (B) The *K_D_* value against sialic acid Neu5Gc was calculated to be 1 μM. Red dots represent dilution concentrations. Blue dots represent the best-fit curve. (C) *K_m_* and *V*_max_ of SiaB against sialic acids Neu5Ac (top) and Neu5Gc (bottom) measured using surface plasmon resonance (Table 1). (D) Illustration showing the principle of the SPR assay to measure SiaB protein activity. SiaB protein was anchored through amine coupling onto a layer of gold containing dextran ligand. Both CTP and Neu5Ac or Neu5Gc are injected into the flowcell chamber. CTP binds to the active site of SiaB followed by Neu5Ac/Neu5Gc binding to form the CMP-Neu5Ac-Neu5Gc complex released from SiaB. The *K_D_* value against CTP (not shown) was found to be >200 μM.

There are several candidate enzymes in the sialic acid NTHi catabolic pathway that could be responsible for this Neu5Ac bias, such as the CMP-sialic acid synthetase (SiaB), which activates sialic acid by adding CMP to form the nucleotide sugar required for enzymatic addition of sialic acid to LOS (49), or the CMP-sialyltransferases SiaA, LsgB, Lic3A, and Lic3B, all of which add sialic acid as the terminal sugar of LOS (40, 50) (Fig. 1). We therefore purified SiaB, which activates these sialic acids to the CMP-sugar form, thus allowing their incorporation into LOS, to determine if the bias for Neu5Ac was occurring at this activation step. Surface plasmon resonance (SPR) analysis was used to determine the SiaB dissociation constant (*K_D_*) values for Neu5Ac and Neu5Gc. This analysis revealed a 600-fold-higher affinity of SiaB for Neu5Ac than for Neu5Gc (Table 1; see also Fig. 4A and B). Having established this substrate binding preference, we devised a novel, on-chip SiaB activity assay using SPR to obtain full enzyme kinetics of SiaB against both Neu5Gc and Neu5Ac. An illustration of the SPR-based kinetic assay is shown in Fig. 4D. These data revealed a 4,000-fold-higher catalytic efficiency (*k*_cat_/*K_m_*) of SiaB for Neu5Ac than for Neu5Gc and thereby revealed that the preferential incorporation of Neu5Ac into LOS (Table 1) is due to more efficient activation with respect to the CMP sugar by SiaB.

**TABLE 1:** Comparison of the CMP-Neu5Ac synthetase enzyme kinetic parameters of NTHi 2019 from this study with those of other organisms and with data from Bravo et al. (56) and from Mizanur and Pohl (57)^*a*^

Source organism (MW, Da)	*K_m_* (μM)	*V*_max_ (μM/min)	*K*_cat_/*K*_m_ (μM^−1^ min^−1^)	Source or reference
NTHi strain 2019 (24,800)				
Neu5Ac	0.143 × 10^−3^ ± 0.012	1.8 ± 0.6	315	This study
Neu5Gc	85.6 × 10^−3^ ± 5.3	0.26 ± 0.04	0.076	This study

Clostridium thermocellum (26,000)				
Neu5Ac	130 ± 10	9 ± 1	1.0	57
Neu5Gc	160 ± 10	7 ± 1	0.6	57

Pasteurella haemolytica (43,000)				
Neu5Ac	1,820 ± 200	197 ± 29	NA	56

a *K_m_* for the donor molecule CTP (not shown) was measured to be 200 μM. MW, molecular weight; NA, not assessed.

### The presence of Neu5Gc on the surface of NTHi leads to increased opsonophagocytic killing.

**FIG 5:**
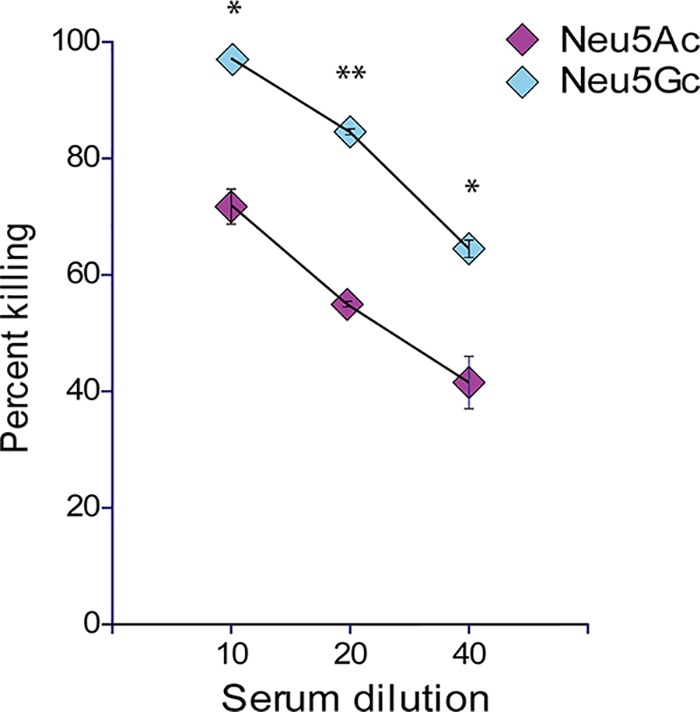
Anti-Neu5Gc-dependent opsonophagocytic killing of NTHi. NTHi strain 2019 was grown with either Neu5Ac (red diamonds) or Neu5Gc (blue diamonds) as a sole carbon source, and opsonophagocytic killing assays were carried out using affinity-purified human anti-Neu5Gc IgGs. Percent killing analyses were carried out by comparisons to a complement-only control. *, *P* = <0.05; **, *P* = <0.005 (calculated using Student's *t* test).

In order to examine if incorporation of Neu5Ac or Neu5Gc into NTHi LOS leads to differences in killing using an anti-Neu5Gc antibody, we grew NTHi strain 2019 in chemically defined RPMI media containing either Neu5Gc or Neu5Ac. Opsonophagocytic killing assays of these strains performed with affinity-purified, human anti-Neu5Gc monospecific antibodies demonstrated that presentation of Neu5Gc on NTHi LOS resulted in a statistically significant increase (*P* = <0.05 at all serum dilutions [Student's *t* test]) in killing by those antisera at all antibody dilutions (Fig. 5).

## DISCUSSION

Recent work has demonstrated a higher incidence of cancer in Neu5Gc-fed mice that have also generated an anti-Neu5Gc immune response (38). It was also proposed that generation of these anti-Neu5Gc xenoautoantibodies in humans derives from an immune response to NTHi displaying Neu5Gc on LOS on the bacterial cell surface early in life (39). These findings support the idea of the importance of understanding the molecular basis of Neu5Gc display on NTHi LOS. As dietary Neu5Gc is transitorily present and relatively low in concentration compared to the abundant Neu5Ac present in the human body, we hypothesized that there may be a bias in NTHi favoring incorporation of Neu5Gc as the terminal sugar on LOS.

Our data on utilization of Neu5Gc and Neu5Ac as carbon sources show that there is no bias in uptake and catabolism of these sugars. This observation is consistent with the results of a previous study that showed that the sialic acid transporter SiaP has a minor preference for binding Neu5Ac versus Neu5Gc (48), but this is likely not the major factor in NTHi presenting Neu5Ac over Neu5Gc on LOS. Our studies examining the relative levels of incorporation of Neu5Ac and Neu5Gc into LOS revealed a bias against the incorporation of Neu5Gc into LOS. Our data support the hypothesis that this preferential incorporation occurs at a crucial step in the NTHi LOS sialylation pathway, where CMP is added to the free sialic acids by SiaB to form the activated CMP-sialic acid required for incorporation into NTHi LOS.

Homologues of CMP-Neu5Ac synthetase SiaB are found in eukaryotes and prokaryotes (51). CMP-sialic acid synthetases have been well studied and characterized in a range of pathogenic bacteria such as Neisseria meningitidis (52), Escherichia coli (53), Streptococcus agalactiae (54), Haemophilus ducreyi (55), Pasteurella haemolytica (now Mannheimia haemolytica) (56), and Pasteurella multocida (52) and in nonpathogenic species such as Clostridium thermocellum (57). Here we found a marked substrate preference for Neu5Ac over Neu5Gc by SiaB of NTHi, indicating exquisite adaptation to the Neu5Ac sialic acid produced by the human host. The only other CMP synthetase enzymes that have been analyzed kinetically for Neu5Ac or Neu5Gc preferences are those from P. haemolytica (56) and C. thermocellum (57). Neither of these enzymes shows a preference for Neu5Ac or Neu5Gc substrates (see Table 1). P. haemolytica is a respiratory pathogen infecting mostly domestic cattle (cows and sheep, both of which are able to produce both Neu5Ac and Neu5Gc). Nonpathogenic C. thermocellum is an anaerobic thermophilic bacterium which lives in a wide variety of habitats, including the human gut. As neither of these organisms is human adapted, it would be expected that they would not contain a SiaB homologue that favors Neu5Ac over Neu5Gc, as they are likely to encounter both these sugars in their niches. In contrast, our demonstration that NTHi has evolved to use either Neu5Ac or Neu5Gc as a carbon source to maximize diversity in nutrient-restricted environments such as the human host but shows preferential utilization of human-exclusive sialic acid Neu5Ac in LOS biosynthesis is evidence of NTHi-human coevolution. NTHi has evolved to avoid the incorporation of Neu5Gc into LOS, and this has likely been driven by immunoevasion of the human immune response to Neu5Gc.

There have been many studies of the mechanisms used by human-adapted pathogens to acquire and utilize nutrients that are abundant in the host, for example, acquisition of iron from human transferrin (58–60) and hemoglobin (61); acquisition of heme from human hemoglobin-haptoglobin (61), complex hemoglobin (62), and myoglobin (63); and utilization of NAD (64–66). Many human-adapted bacterial pathogens also coat themselves with human-derived molecules in order to evade the immune system; for example, Campylobacter jejuni adds GD1a-like epitopes in LOS, which mimic human gangliosides (67); Neisseria meningitidis expresses LOS with terminal lacto-*N*-neotetraose (68, 69), the same structure present on paragloboside, the precursor to the ABH antigens on human blood cells; and Helicobacter pylori lipopolysaccharide (LPS) contains fucosylated glycans that mimic the Lewis X and Y antigens also found on human blood cells (70). A number of human-adapted bacterial pathogens also specifically target Neu5Ac in preference to Neu5Gc; for example, Streptococcus pneumoniae preferentially recognizes and utilizes Neu5Ac (71), and the toxin produced by Salmonella enterica serovar Typhi has a much higher affinity for cells with Neu5Ac-containing glycan structures than for those containing Neu5Gc (72). We recently reported that two major adhesins of NTHi, the HMW1/2 proteins, preferentially bind Neu5Ac sialic acid-containing glycans over Neu5Gc-containing glycans (73), demonstrating that NTHi preferentially binds glycans present in the human host. Our findings reported in this study indicate the molecular basis for a further host adaptation in NTHi. While sialic acid uptake and the presence of catabolic enzymes allow NTHi to utilize either Neu5Ac or Neu5Gc as a carbon source for growth, the exquisite preference of SiaB for Neu5Ac allows NTHi to limit the level of Neu5Gc on the cell surface and to avoid killing by the highly abundant anti-Neu5Gc antibodies found in human serum. The presence of human antibodies specific for Neu5Gc-containing structures is likely the driving force for this adaptation and demonstrates that NTHi is highly adapted to the human host.

## MATERIALS AND METHODS

### Bacterial strains, media, and culture conditions.

NTHi strain 2019 is a human clinical isolate (74). NTHi was grown on brain heart infusion (BHI) agar (Oxoid, United Kingdom) supplemented (sBHI) with 1% (vol/vol) hemin (Sigma) and 20 μg/ml NAD^+^ (Sigma) or on chocolate agar prepared by adding 40 ml of fresh defibrinated horse blood to 400 ml of blood agar base no. 2 (Oxoid) and heating until the blood lysed. NTHi cultures were incubated at 37°C for 16 h in the presence of 5% (vol/vol) CO_2_. Sialic acid sole carbon source growth experiments were carried out in chemically defined media as described previously (47). For sialic acid-free chemically defined media, an RPMI 1640 (Sigma) formulation was used and supplemented as described previously (46). Escherichia coli BL21(DE3) was grown on Luria-Bertani (LB) broth (Oxoid), with 100 μg/ml ampicillin (Sigma) where appropriate, at 37°C with 200 rpm shaking.

### Growth experiments with sialic acid as the sole carbon source.

NTHi strain 2019 was first grown on sBHI agar for 16 h as described above. Bacterial plate growth was collected, suspended in sBHI broth, and centrifuged at 5,000 rpm for 5 min to collect the cell pellet. The cell pellet was washed and grown in sialic acid-free chemically defined RPMI 1640 medium supplemented with 1% (vol/vol) hemin and 20 µg/ml NAD (sRPMI) as described previously (47). Optical density (OD) was normalized for cultures with a starting growth level of an OD at 600 nm (OD_600_) of 0.05. Cultures were supplemented with 100 μM sialic acid Neu5Ac or Neu5Gc (Inalco), 20% glucose (Sigma), or sodium pyruvate (Sigma) as the sole carbon source, where applicable. Growth was measured as a function of OD_600_ over 12 h at 37°C.

### NTHi LOS purification and preparation.

NTHi strain 2019 was first grown on chocolate agar for 16 h as described above and then restreaked onto sRPMI medium plates supplemented with differing molar ratios of the sialic acids Neu5Ac and Neu5Gc. Plates were then incubated for 16 h at 37°C in the presence of 5% (vol/vol) CO_2_. Bacterial growth was collected and resuspended in nuffer A (60 mM Tris base, 10 mM EDTA, 2% SDS, pH 6.8). LOS was purified using a proteinase K/hot phenol method as described previously (75, 76). Purified LOS was resuspended in ultrapure chromatography-grade water (Sigma), frozen at −80 °C, and lyophilized overnight using a freeze dryer.

### NTHi LOS sialic acid analysis.

Following quantification of the dry weight of each freeze-dried purified LOS sample, a 1 mg/ml stock of each sample was prepared in ultrapure chromatography-grade water. A subsample (80 µl) was subjected to acid hydrolysis using trifluoroacetic acid (3.35 M). At the end of the reaction, the sample volume was dried under vacuum and the residue reconstituted using an internal standard, ketodeoxynonulosonic acid (100 µl, 100 µM). The analysis was carried out at the Australian Proteome Analysis Facility (APAF; Macquarie University, Sydney, Australia) using an instrument incorporating high-pH anion-exchange chromatography with pulsed amperometric detection (HPAEC-PAD) fitted with a CarboPAC PA1 guard column (2 by 50 mm) connected to a CarboPAC PA1 column (2 by 250 mm) held at 25°C as described previously (77). The sample (20 µl) was injected into the HPAEC-PAD instrument and analyzed using a separation gradient of 60 to 300 mM sodium acetate–sodium hydroxide (100 mM), at a flow rate of 0.5 ml/min. The analytes detected were quantified using internal calibration as described previously (78).

### NTHi 2019 CMP-Neu5Ac synthetase (SiaB) protein expression and purification.

*siaB* from NTHi strain 2019 was amplified using Thermococcus kodakaraensis (KOD) polymerase according to the instructions of the manufacturer (TaKaRa Bio) and primers siaB-F (5′-GACGACGACAAGATGAAAATAATAATGACAAGAATTGCAATT-3′) and siaB-R (5′-GAGGAGAAGCCCGGGAATTCTTTTGAAATTAAACTTTCGG-3′) and was cloned into overexpression vector pET51-Ek/LIC (Merck Millipore) according to manufacturer’s instructions so as to be in-frame with an N-terminal 6×His tag. (Sequences in each primer used for ligation-independent cloning [LIC] are underlined.) Clones were confirmed by sequencing using BigDye Terminator 3.1 (Thermo Fisher) according to the manufacturer’s instructions. The resulting construct was designated pET51::2019*siaB*. SiaB overexpression was induced with 0.1 mM isopropyl β-d-1-thiogalactopyranoside (IPTG) (Sigma), and the cultures were grown at 37°C with shaking at 200 rpm for 4 h. Overexpressed cultures were pelleted at 5,000 rpm for 15 min at 4°C, and the pellet was then resuspended in lysis buffer (50 mM NaH_2_PO_4_, 300 mM NaCl, pH 7) containing protease inhibitor cocktail (Roche), lysozyme (0.2 mg/ml), and DNase (10 µg/ml). Cells were lysed using a TissueLyser (Qiagen; five rounds of 1 min at 50 oscillations^−1^ s^−1^/1 min on ice). Lysate was clarified by centrifugation at 10,000 rpm for 30 min. Supernatant was decanted and mixed with 1/3 volume of lysis buffer containing 20 mM imidazole (Sigma) and was applied to a column containing Talon resin (Clontech). The column was washed with lysis buffer containing 20 mM imidazole (5 column volumes), and SiaB was eluted with lysis buffer containing 500 mM imidazole. Following analysis of fractions by SDS-PAGE, fractions containing pure SiaB were pooled and dialyzed against lysis buffer (5 liters) overnight at 4°C and were concentrated using Amersham centrifugal concentrators (Merck Millipore) according to the manufacturer’s instructions. Protein concentration was determined using a bicinchoninic acid (BCA) protein assay (Thermo Fisher).

### Enzyme assay and kinetics of SiaB using surface plasmon resonance (SPR).

The enzyme assay used in this study was modified from methods previously described (79). Purified SiaB was immobilized onto a Series S CM5 sensor chip by amine coupling. Briefly, the carboxy dextran surface was prepared by injection of NHS (*N*-hydroxysuccinimide)/EDC [1-ethyl-3-(3-dimethylaminopropyl)-carbodiimide)] followed by the SiaB protein at 100 µg/ml. The protein was cross-linked by the addition of ethanolamine. Flow cell 1 served as a reference cell and was prepared under the same conditions but without immobilized protein. CTP and sialic acids (Neu5Ac and Neu5Gc) were diluted in buffer (20 mM MgCl_2_, 0.2 M MOPS [morpholinepropanesulfonic acid], pH 8.1) (57) at concentrations ranging from 100 µM to 0.05 nM. Single-cycle and multicycle kinetic experiments were performed at 37°C using a Biacore T100 system (GE Healthcare). Affinity constants (*K_D_*) for CTP, Neu5Ac, and Neu5Gc were determined by the use of analysis software. Enzyme kinetic parameters *K_m_*, *V*_max_, and *K*_cat_/*K_m_* were calculated for Neu5Ac and Neu5Gc (Table 1).

### Opsonophagocytic killing assays.

Opsonophagocytic killing assays were carried out as described previously (80). NTHi strain 2019 was grown in supplemented RPMI medium with either Neu5Ac or Neu5Gc, with the two strains being compared in the same experiment. Experiments were carried out in duplicate using a 1:10 to 1:40 dilution of primary anti-Neu5Gc IgG antibody that had been subjected to affinity purification from human intravenous immunoglobulin (IVIG) (31). Percent killing at each dilution was calculated by determining the ratio of the bacterial colony count at each dilution to that of the complement-only serum control.
